# Roles of Surfactants in Oriented Immobilization of Cellulase on Nanocarriers and Multiphase Hydrolysis System

**DOI:** 10.3389/fchem.2022.884398

**Published:** 2022-03-23

**Authors:** Zhiquan Wang, Chunzhen Fan, Xiangyong Zheng, Zhan Jin, Ke Bei, Min Zhao, Hainan Kong

**Affiliations:** ^1^ School of Life and Environmental Science, Wenzhou University, Wenzhou, China; ^2^ State and Local Joint Engineering Research Center for Ecological Treatment Technology of Urban Water Pollution, Wenzhou, China; ^3^ Zhejiang Provincial Key Lab for Water Environment and Marine Biological Resources Protection, Wenzhou, China; ^4^ School of Environmental Science and Engineering, Shanghai Jiao Tong University, Shanghai, China

**Keywords:** cellulase, surfactants, nanocarriers, reversed micelle system, oriented immobilization

## Abstract

Surfactants, especially non-ionic surfactants, play an important role in the preparation of nanocarriers and can also promote the enzymatic hydrolysis of lignocellulose. A broad overview of the current status of surfactants on the immobilization of cellulase is provided in this review. In addition, the restricting factors in cellulase immobilization in the complex multiphase hydrolysis system are discussed, including the carrier structure characteristics, solid-solid contact obstacles, external diffusion resistance, limited recycling frequency, and nonproductive combination of enzyme active centers. Furthermore, promising prospects of cellulase-oriented immobilization are proposed, including the hydrophilic-hydrophobic interaction of surfactants and cellulase in the oil-water reaction system, the reversed micelle system of surfactants, and the possible oriented immobilization mechanism.

## Introduction

Bioethanol, as a renewable, economically affordable, and environmentally safe energy material, will gradually become a substitute for fossil fuels. It has far-reaching research significance and application value for the development of a sustainable energy strategy (Karimi et al., 2021; Zeng et al., 2021; Ziaei-Rad et al., 2021; Suhartini et al., 2022). Due to competition with food supply in the first generation of bioethanol production, lignocellulose, a non-starch material, has become an important raw material for bioethanol production (Alonso et al., 2019; Maia et al., 2020; Winarni et al., 2020). Adsorption of cellulases onto lignin has been considered as the major factor in retarding enzymatic cellulose degradation of lignocellulosic biomass (Djajadi et al., 2018). Hydrophobic interaction, electrostatic interaction and hydrogen bonding have been regarded as the cause of the nonproductive binding of cellulases to lignin (Djajadi et al., 2018; Li et al., 2020; Song et al., 2020). A natural “biodegradable barrier” of lignin cell walls which are connected in a strong, yet resilient network under the action of covalent and non-covalent bonds render the cellulose inaccessible (Mnich et al., 2020; Chu et al., 2021). Therefore, to reduce the recalcitrance of lignocellulosic biomass to biochemical degradation, pretreatment methods have been developed to break down the lignin-hemicellulose-cellulose matrix and increase the enzyme accessibility of the cellulose scaffold (Jiang et al., 2017a; Jia et al., 2018; Rocha-Martin et al., 2018).

In general, lignin-derived inhibition is the major physical obstacle restricting the enzymatic hydrolysis of cell wall polysaccharides (Leonidas et al., 2019; Zheng et al., 2021). More importantly, the non-specific binding of free cellulase on lignocellulosic substrates may account for the low rate of hydrolysis at the action mechanism level during enzymatic hydrolysis. Some enzymes remain free after the enzymatic hydrolysis of lignocellulosic substrates, while non-specific binding to the residual substrates also prevents the efficient recycling of cellulase (Rahikainen et al., 2011; Kellock et al., 2017; Bhawna et al., 2020). Moreover, the utility of cellulases has been limited due to their low operational stability, high costs, and poor reutilization when used in the native form (Yang et al., 2017).

To overcome these barriers, immobilization is usually used to improve enzyme stability and even activity or selectivity when properly designed, which can also facilitate the reuse of enzymes and effective cost of catalytic processes (Mita and Eldin, 2014; Li et al., 2016; Mehta et al., 2016; Xu et al., 2016; Zhang et al., 2016). The characteristics of various immobilization methods of enzymes is summarized in Table 1. Cellulases represent a large group of enzymes from various organism and with different substrate specificity, biophysical properties, etc. The immobilization behavior is different depending on the enzyme or enzyme mixture investigated. During the immobilization process of cellulase, the structure and properties of carrier materials have significant effects on the performance of the immobilized enzyme (Kalantari et al., 2013; Li et al., 2018). The size of the carriers plays an important role in determining the activity of the immobilized enzyme owing to the inverse relationship between the carrier size and enzyme loading. Thus, large carrier size decreases enzyme activity in general (Valencia et al., 2010), and a reduction in the size of the carriers results in a higher surface area for enzyme binding (Malar et al., 2018; Malar et al., 2020). For the immobilization of cellulase, the smaller size of the surface pore should be kept lower than that of the cellulase macromolecule (6–20 nm), which can further reduce the internal and external diffusion resistance in the heterogeneous system (DiCosimo et al., 2013; Santos et al., 2015). Therefore, nanocarriers are widely used in the immobilization of enzymes because of their unique properties, such as large specific surface area to volume ratio (Cao et al., 2016; Roth et al., 2016; Malar et al., 2020).

**TABLE 1:** The characteristics of various immobilization method of enzymes.

Methods		Mechanisms	Characteristics	References
Adsorption	Physical	Adsorbed on the carriers	Active center of the enzyme is not easy to be destroyed, and not obvious structure change occurs	Gao et al. (2009)
	Ionic	Combined with water-insoluble carrier containing ion-exchange group by electrostatic force	Structure and amino acids of the active center rarely change, and the higher activity immobilized enzyme can be obtained	Sui et al. (2015)
Encapsulation		Mixed with polymer monomer and further embedded in the polymer	It is not necessary to combine with amino acid residues of enzyme protein, and rarely change the spatial conformation of enzyme	Singh et al. (2020)
Covalent binding		Covalently bonded to the water-insoluble carrier	Enzyme molecules are firmly connected with the carrier, the structure of the enzyme protein is often changed, resulting in the damage of the active center of the enzyme	Ghasemi et al. (2021)
Cross-linking		Bifunctional reagent or multifunctional reagent is used to form covalent bond between enzyme molecules	Combined with adsorption or encapsulation method, the activity of immobilized enzyme can be increased and the reinforcement effect can be achieved	Ouyang et al. (2020)
Cross-linked enzyme aggregates (CLEAs)		Covalently bound by cross-linking agent to keep the supramolecular structure and activity	Carrier free immobilization, good stability, low cost, large activity per unit volume, and high space efficiency	Xu et al. (2020)
Co-immobilization		Different enzymes are immobilized in the same carrier at the same time	Several kinds of enzymes and cells with different functions work together in the same system	Qiu et al. (2021)
Oriented immobilization		Specific site of enzyme connects with carrier and the active site faces outsid	It is beneficial for the substrates to enter into the active site of the enzyme and can significantly improve the activity of the immobilized enzyme	Zhou et al. (2021)

Moreover, the immobilization of cellulase has been achieved based on physical adsorption, covalent binding, or affinity interactions (Zang et al., 2014; Hosseini et al., 2018; Zhang and Hay, 2019), including carrier-binding, microemulsion-based organo-gels (MBGs), ultrasonic encapsulation, crosslinking, entrapment, glutathione-labeling, and chelation (Mroczkiewicz et al., 2012; Nicoletti et al., 2015). However, enzymes often display drastically lower activity in organic solvents than in water, and the water layer on the molecular surface of enzymes determines their activity in organic media (Zhang et al., 2012). Therefore, among several approaches to resolve the challenges, one of the most effective methods is immobilization of the enzymes within an aqueous microenvironment in the organic solvents. Microemulsions formed by amphiphilic surfactants have been widely reviewed as effective media for the immobilization of enzymes in hydrophobic solvents (Itabaiana et al., 2014; Rajnish et al., 2021; Savic et al., 2021). The MBGs method based on microemulsions has been used to form matrices for enzyme immobilization to achieve enzymatic catalysis in nonconventional medium as they appear to be rigid and stable for a long time, even within the reaction solution (Zhang et al., 2012). Therefore, the MBGs method has unique advantages of improving the chemical stability of immobilized enzymes and maintaining high catalytic activity (Pavlidis et al., 2010; Itabaiana et al., 2014). It is clearly that the surfactants play an important role in the preparation of nanomaterials (Lou et al., 2017; Bao et al., 2019; Ortiz-Martínez et al., 2019; Alexander et al., 2020).

The surfactants have been widely used for the preparation of nanocarriers as shown in Table 2, forming the nano-template by micelles and emulsions of surfactants is a common method that can greatly reduce the surface tension of the solvent and change the interface composition and structure (Carter and Puig-Sellart, 2016; Bao et al., 2019). Desirable nanostructured materials can be produced because of the special nanoreactors formed by surfactant micelles and the oriented alignment characteristics of surfactants in solution, such as the Langmuir-Blodgett (LB) membranes and liposomes (Lok Kumar et al., 2014; Gutierrez et al., 2016). Furthermore, the non-ionic surfactants can significantly enhance cellulose hydrolysis, thus reducing enzyme loading (Lou et al., 2017; Bao et al., 2019). However, inhibitory effects have been observed with the addition of amphoteric, anionic, and cationic surfactants (Lou et al., 2017; Bao et al., 2019). Moreover, the loss of enzyme activity during immobilization is a notable problem; the structural distortion caused by the strong enzyme-support interactions may produce steric hindrances and catalytic cleft blockage (Carlsson et al., 2014; Suárez et al., 2018). Although a large dose of original cellulase is added for a higher load of immobilized enzyme to improve the activities of the immobilized enzyme, no significant improvement in enzymatic activity has been observed due to the random and inhomogeneous combination of the nanocarriers and cellulase molecules (Nakayama et al., 2009). Oriented immobilization, as a specific binding method, can effectively prevent the nonproductive combination of enzymes and nanocarriers, which further improves the immobilization and hydrolysis efficiency. The reversed micelles formed by surfactants have been successfully used in the preparation of oriented-immobilized lipase when their concentration exceeds the critical micelle concentration (CMC) (Fan et al., 2016). To date, few studies have reported the oriented immobilization of cellulase. Therefore, this review mainly focuses on the important roles of surfactants in the immobilization of cellulase, mainly including the preparation of nanocarriers and cellulase hydrolysis. Moreover, a novel insight into the oriented immobilization of cellulase in a surfactant reversed micelle (SRM) system was discussed and found to have promising prospects.

**TABLE 2:** Applications of surfactants in preparing nanomaterials.

Applications	Types	Characteristics	References
Nanomaterials	Metallic nanoparticles	It is usually prepared in the reversed micelles and microemulsions system	Kawasaki, (2013)
	Semiconductor nanoparticles	It is prepared in the reversed microemulsions system, including the oxides, sulfides, and selenides etc.	Anjum et al. (2019)
	Organic nanoparticles	It includes organic drug nanoparticles and polymer nanoparticles, which can be prepared in microemulsions system	(Li, Kawakami, and Hiramatsu, 2003)
	Nanowires	It can be prepared by the templates from micelles, liquid crystals, vesicles formed by the surfactants	Xu et al. (2010)
	Porous nano-materials	Surfactants can be the structure directing agent of mesoporous materials	Carrillo et al. (2011)
	Nano-films	It mainly includes Langrnuir-Blodgett (LB) film and Molecular-Deposition (MD) film	Shil et al. (2017); Lai et al. (2020)
	Nanocomposites	Organic polymer was encapsulated on inorganic nanoparticles in inverse microemulsion system	Al-Shemmari et al. (2014)
Methods	Template-directed synthesis	The electrostatic attraction, hydrogen bond and Van der Waals force between surfactant molecules and nano materials are used for the formation of special micelle structures, which can further used as the synthesis templates of nano materials	Xu et al. (2010); Kayhomayun et al. (2020)
	Microemlusion method	When the amount of surfactant and polar organic matter is large, the microemulsion can be obtained, which can be used as a microreactor for synthesizing nanomaterials	Anjum et al. (2019); Cui et al. (2019)
	Hydrothermal synthesis	Surfactants are mainly used as auxiliary materials	Cui et al. (2019)
	Sol-gel method	The transparent sol is formed by hydrolysis and condensation reaction, and gradually gelatinization. After drying and heat treatment, nanomaterials can be obtained	Hassanzadeh-Tabrizi et al. (2016)
Surface modification	Physical and chemical properties	Surface adsorption and chemical reactivity of surfactants can modify the surface of nanoparticles	Chaudhary et al. (2014)
	Interfacial modification of nanofilms	Hydrophilicity or lipophilicity of surfactants can be used to modify the interface of nanofilms	Kovalchuk, (2015)
Effects	Dispersion of nanoparticles in media	Prevent particle agglomeration	Fiorati et al. (2021)
	Functional effects on nanoparticles	Improve the compatibility and affinity between polymer materials and inorganic materials	You et al. (2019)

## Effects of Surfactants on Nanocarriers

### Preparation of Nanocarriers Based on Surfactants

The basic physical and chemical properties of surfactants, such as micelle formation, dispersing, emulsifying, and solubilizing, have made them widely useful in the field of nanotechnology (Yang et al., 2017). Several ordered aggregations formed by the surfactants are used as nano-templates for the preparation of nanocarriers, such as micelles and reversed micelles. The process can greatly reduce the surface tension of the solvent and change the interface composition and structure (Bao et al., 2019). For the preparation of nanocarriers, surfactant micelles are the microreactors of nanocarriers during the preparation process, and the morphology of microreactors is controllable because of the amphiphilic characteristics of surfactants, which have been used for the preparation of desirable nanostructured carriers (Yiamsawas et al., 2017). For instance, hydrophilic surfactants are often used for the preparation of spherical nanocarriers because of their dispersibility in water (Luan and Ramos, 2010). Similarly, the reversed micelles of surfactants can effectively define the particle size and reaction microenvironment in the water, providing a nanoscale reaction space. It has been widely used because the aggregates self-assembled by surfactant molecules can be used to synthesize ordered mesoporous materials with a simpler operation and more uniform channel distribution (Bao et al., 2017; Bao et al., 2019).

### Surface Modification of Nanocarriers in the Surfactant System

Surfactants can also change the surface properties of nanocarriers, such as their morphology, magnetic properties, dispersion, and catalytic performances (Asghar et al., 2016; Wei et al., 2018; Lopes et al., 2019; Alexander et al., 2020). This modification may result in a new structure with new surface activity due to the combination of hydrophilic groups of surfactants and surface groups of nanocarriers. For example, the use of surfactants of decylamine and cetyltrimethylammonium bromide can provide an easy and effective way to change the functionality of cellulose nanocrystals with a hydrophobic polylactic acid matrix and to evaluate the effects of surface chemistry on the reinforcement mechanisms (Orellana et al., 2018). Meanwhile, the presence of surfactants can make nanocarriers more difficult to re-agglomerate by reducing the surface energy and form a steric hindrance effect (Wang M. et al., 2013; Tan et al., 2019), the surfactants are coated on the surface of the nanocarriers to form a space barrier layer, the hydrophilic group faces outward and the hydrophobic group faces inward, so that the agglomeration of the particles is avoided.

## Effects of Nanocarriers on Immobilization of Cellulase

The structure and properties of carrier materials have great influence on the properties of immobilized cellulase, such as internal geometry (e.g., flat surfaces or thin fibers), specific surface area, superficial activation degree, mechanical resistance, and pore diameter (Santos et al., 2015; Begum et al., 2019; Malar et al., 2020). Meanwhile, partitioning and mass transport limitations may yield spatial variations in local reaction rates in porous materials (Neira and Herr, 2017). Therefore, to improve the stability and catalytic activity of immobilized cellulase, various materials, such as chitin, chitosan, nylon, and polyvinyl alcohol, have been widely used as carriers (Cherian et al., 2015; Priydarshani et al., 2018).

The physical effects of nanocarriers on immobilized cellulase are as follows: 1) The pore size and effective surface area of the nanocarriers. Not all porous carriers can be used for immobilization of cellulase due to the limitation of pore size, which should be larger than or equal to that of the cellulase to reduce steric hindrance. The effective surface area occupied by the enzyme determines the maximum load of the immobilized cellulase (Santos et al., 2015). When a stable surface area is maintained, the amount of immobilized or absorbed cellulases is related to the pore size because the pore diameter determines the size of the protein that can be immobilized on that carrier (Teresa et al., 2009; Webster et al., 2015); 2) the number of carrier-bound active groups (CAGs) is another key factor controlling the enzyme-carrier multi-interaction (Cristina et al., 2011; Santos et al., 2015); 3) the size of carriers plays a very important role in the preparation of immobilized cellulase, in that a smaller carrier size with larger specific surface area will be better for the cellulase immobilization load, and the higher surface porosity of the carriers providing numerous binding sites for cellulase is one of the most important factors influencing the activity of immobilized cellulase (Chen et al., 2010; Santos et al., 2015; Malar et al., 2020); 4) the mechanical properties of the carriers need to be controlled considering the final configuration of the reactor. If the reactor is a fixed-bed reactor, such as inorganic supports like porous glass, silicates, it should possess very high rigidity to withstand high pressures without pressure problems, but the situation is different if a stirred-tank reactor is used (Cristina et al., 2011; Santos et al., 2015); 5) after the cellulase penetrates the carriers, the internal morphology of carriers will determine the possibility of obtaining a very intense or very limited enzyme-carrier interaction (Santos et al., 2015). When the diameter of the carriers is smaller than that of the enzyme, it is difficult to obtain an intense enzyme-carrier interaction (Cristina et al., 2011), but if the carriers have sufficiently large internal surfaces, it is possible to get an intense interaction with a similar flat surface (e.g., agarose beads, porous glass, or silicates) (Malar et al., 2018).

**FIGURE 1:**
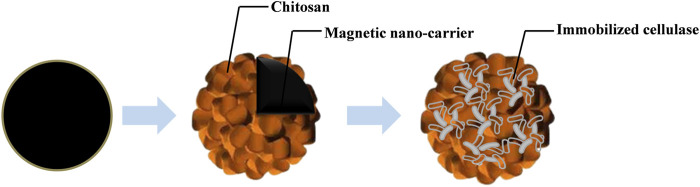
Schematic diagram of immobilized cellulase on a magnetic nanocarrier.

In particular, the special superparamagnetism of magnetic nanocarriers has attracted increasing interest as they allow easy recycling and separation of catalysts and biomolecules from high-viscosity liqueurs and high-solid-content broths. This unique characteristic has been well-applied to immobilization of cellulase, and a better hydrolysis efficiency and recycling feasibility have been observed (Alftrén et al., 2014; Cao et al., 2016; Cipolatti et al., 2014; Xing et al., 2015). During immobilization of cellulase, magnetic chitosan microspheres (C-MNPs) are frequently used as carriers because of their significant biological (i.e., biodegradable, biocompatible, bioactive) and chemical properties (polycationic, hydrogel, contains reactive groups, such as -OH and -NH_2_). Moreover, the hydrophilic properties of the C-MNPs play an important role in the preparation of oriented-immobilized cellulase based on the SRM system. The conventional immobilization of cellulase molecules on a single magnetic nanocarrier is simple, the chitosan was usually first coated on the magnetic nanocarriers for further combination with cellulase (Figure 1). Subsequently, the combined material based on Fe_3_O_4_ nanocarriers have received extensive attention in cellulase immobilization to improve enzyme activity, loading, and stability because of their low toxicity, biocompatibility, and easy synthesis (Jordan et al., 2011). Magnetite nanocarriers coated with silica and modified by organic-silanes, biocompatible, and with hydrophilic properties, are promising for cellulase immobilization.

**FIGURE 2:**
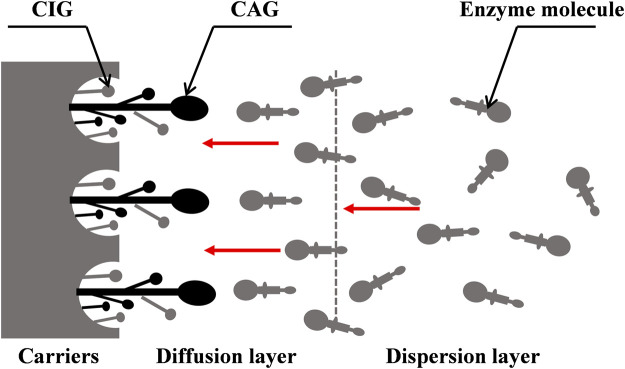
Binding schematic diagram of enzyme and carrier caused by the chemistry properties during the immobilization process. CIGs means the carrier-bound inert groups and CAGs means the carrier-bound active groups; The larger end of enzyme molecule stands for the catalytic domain and the other end stands for the adsorption domain.

The binding sites of enzymes on the surfaces of carriers depend on the chemical properties of the carriers. For non-covalent immobilization, the chemical structure of the skeleton and surface determines the applicability of carriers. The functional groups play a key role in the activity, stability, and selectivity of the enzyme, and the size, charge, polarity, and hydrophilicity/hydrophobicity of groups can affect their binding functions (Watanabe et al., 2010). Different properties of the ionic groups on the surfaces of carriers may result in different enzyme activities and further determine the structure of immobilized cellulase (Santos et al., 2015; Berlin et al., 2016; Frančič et al., 2016; Hui et al., 2016; Zhou et al., 2018). The chemistry properties of enzyme and carrier cause the oriented distribution of catalytic domain of enzyme from dispersion layer to diffusion layer during the immobilization process is shown in Figure 2. In this process, the CAGs directly participate in binding with enzyme molecules, but the carrier-bound inert groups (CIGs) are not directly involved. This interaction inevitably disturbs the maintenance of the natural conformation of the enzyme, leading to structural and functional changes in the enzyme molecules. No obvious stability change has been observed when the newly formed conformation is similar to that of the natural enzyme.

The covalent binding between carriers and catalytic cleft of the enzyme not only causes pore plugging of the surface, but also leads to the drag increment of in-diffusion. Although an initial high dosage of cellulase is added, the inhomogeneous distribution of the carrier surface structure results in the uncontrollable immobilization sites, and ineffective immobilization may lead to a significant loss of enzymatic activity and reduce the accessibility of the substrate to the functional sites. Moreover, the partition and mass transport limitations of nanocarriers may cause spatial variation in local reaction rates and further affect enzymatic hydrolysis (Du et al., 2017; Zeng et al., 2019). The chitosan molecules are mostly used because of the large number of -OH and amino groups (-NH_3_), which are easier to co-precipitate with cellulase (Bindhu and Abraham, 2010; Urrutia et al., 2018; Saha et al., 2019; Mo et al., 2020). Moreover, surface modification is an important strategy for tuning the properties of nanocarriers. Surface modification can either alter the existing property or introduce new properties onto nanoparticles using various agents, such as organ siloxane, N-(3-dimethylaminopropyl)-N′-ethylcarbodiimide hydrochloride (EDC), and carbodiimide, as well as amino silanes, such as 3-aminopropyltriethoxysilane, aminoethyl aminopropyl polydimethylsiloxane, and silica (Chang et al., 2011; Gokhale et al., 2013; Riedel et al., 2017; Malar et al., 2018; Malar et al., 2020).

## Roles of Surfactants on Cellulase Hydrolysis

In most reports about hydrophobic ionic liquids, the enzymes are not dissolved but merely in a dispersed state and therefore regarded as a heterogeneous catalyst. Some hydrophilic ionic liquids can accelerate the dissolution of enzyme molecules, but cause the destruction of the protein secondary structure, leading to the inactivation of the enzyme (Fujita and Ohno, 2010; Moniruzzaman et al., 2010). In pure hydrophilic ionic liquids the enzymes can be dispersed at the monomolecular level. The hydrophilic proteins in almost anhydrous nonpolar solvents form suspensions, whereas proteins with extended hydrophobic surface segments form microemulsions in the same media, greatly reducing the catalytic efficiency of the enzyme (Zuev et al., 2003; Predvoditelev et al., 2010). Nonpolar hydrophobic solvents, such as heptane, octane, and benzene, do not cause the dehydration of biocatalysts. Therefore, the enzyme can maintain its catalytic activity (Muginova et al., 2010). Similarly, the catalytic activity of enzymes can be retained in the surfactant micelle system due to the water-oil amphiphilicity of surfactants (Muginova et al., 2010). Non-ionic surfactants can significantly accelerate the enzymatic hydrolysis of lignocellulose (Qing et al., 2010; Seo et al., 2011b; Eckard et al., 2012; Yiamsawas et al., 2017). For instance, Tween-20 can enhance the specific adsorption of cellulase, and the conversion efficiency of cellulose increased from 9 to 21% within 72 h when a high lignocellulosic substrate was added (Seo et al., 2011a). The prevention of non-productive enzyme adsorption onto lignin is the most widely investigated mechanism for this enhancement (Sipos et al., 2010; Lou et al., 2017). Recently, Djajadi et al. (2018) has proved that the adsorption of cellulases onto lignin substrates is reversible by nature, the reversible adsorption-desorption is existing in the process. But the non-productive adsorption caused by the ineffective combination will occupy large number of catalytic clefts of enzyme molecule which greatly hinder the enzymatic hydrolysis. Non-ionic surfactants can render lignin surfaces more hydrophilic by increasing their polar surface energy component, which can reduce the non-productive adsorption of cellulases onto lignocellulosic substrates caused by the ineffective combination between catalytic clefts of enzymes and lignin substrates (Jiang et al., 2017b), thereby promoting the enzymatic hydrolysis of lignocellulose. However, for the anionic surfactant-cellulase system, the adsorbed surfactants on the surface of cellulase cause a lower negative charge area, which further leads to negative catalytic activity due to the presence of sulfonic acid groups with a higher ionization degree (Yu and Zhang, 2016).

Furthermore, the effect of surfactants on cellulase hydrolysis is related to the concentration of surfactants (Zhou et al., 2015). In the enzymatic hydrolysis process, cellulose molecules are specifically adsorbed by the cellulose-binding domain (carbohydrate-binding module, CBM) and exert a driving force on the enzyme during the hydrolysis of cellulose (Liu et al., 2011; Tomme et al., 2015; Arslan et al., 2016). The adsorption of CBM can increase the cellulase concentration of the substrate surface by promoting the association of enzymes and substrates, but the non-covalent interactions (e.g., hydrogen bonds, electrostatic, and hydrophobic interactions) may lead to a nonproductive combination, because the random combination will occupy the active center of enzyme, resulting in the loss of catalytic activity. Ineffective adsorption can be reduced in the presence of surfactants due to the hydrophobic structure of surfactants, which can interact with the hydrophobic lignocellulosic substrates and form a coating (Kumar and Wyman, 2010; Li et al., 2012). However, contrasting results were obtained when different concentrations of surfactants were added to the enzymatic hydrolysis system. Some studies have suggested that a high concentration of surfactants can inhibit cellulase activity because strong hydrophobic interaction between the surfactant and cellulase can further reduce the effective adsorption of enzymes on cellulose (Wang Z. et al., 2013; Bao et al., 2019). However, the promotion effect of surfactant in enzymatic saccharification was observed in low concentration of lignosulfonate with low molecular weight and good sulfonation, which can be explained that the lignosulfonate can prevent the nonproductive binding of cellulase to lignin substrate, and the formed lignosulfonate-cellulase aggregate can also stabilize and enhance the binding of cellulase to lignin substrate (Lou et al., 2014).

## The Oriented Immobilization of Cellulase in the SRM System

The oriented immobilization of proteins on a solid support can effectively avoid its denaturation and keep its catalytic clefts fully exposed to solution, thus maximally preserving the bioaffinity or bioactivity. Liu and Yu (2016) has summarized the recent advances in oriented immobilization of proteins with a particular focus on antibodies and enzymes. However, the orientated immobilization of enzymes at the solvent interface is never involved. Thereby, the follow-up content will propose a novel method to achieve the oriented immobilization of cellulase in the SRM system.

### Construction of the SRM System

The SRM system has been widely used in the preparation of immobilized enzymes (Dong et al., 2010; Marhuendaegea et al., 2015). The special structure of surfactant molecules caused a water-oil amphipathy with a hydrophobic nonpolar hydrocarbon chain (alkyl) and a hydrophilic polar group (such as -OH, -COOH, -NH_2_, and -SO_3_H) distributed at different ends. In the water-oil (W/O) system, the surfactants are dissolved in the nonpolar organic solvent when a trace of water is provided, and the reversed micelles are formed when the concentration exceeds the CMC (Takagi et al., 2019; Chi et al., 2018). In reversed micelles, the nonpolar groups of the surfactants are exposed to the nonpolar organic solvents, while the polar groups are arranged inside. Therefore, a polar core with the ability to dissolve polar substances in the microreactors is formed. The SRMs are nanoscale aggregates that are formed spontaneously, and the W/O microemulsion with low water content provides a stable thermodynamic system (Tao et al., 2013). According to the hydrophilic-hydrophobic interaction of surfactants and cellulase in the oil-water reaction system, the large number of oil-water interfaces in the system provides a good environment for the catalytic reaction of enzyme molecules (Brady and Jordaan, 2009).

### Mechanism of Oriented-Immobilized Cellulase in the SRM System

**FIGURE 3:**
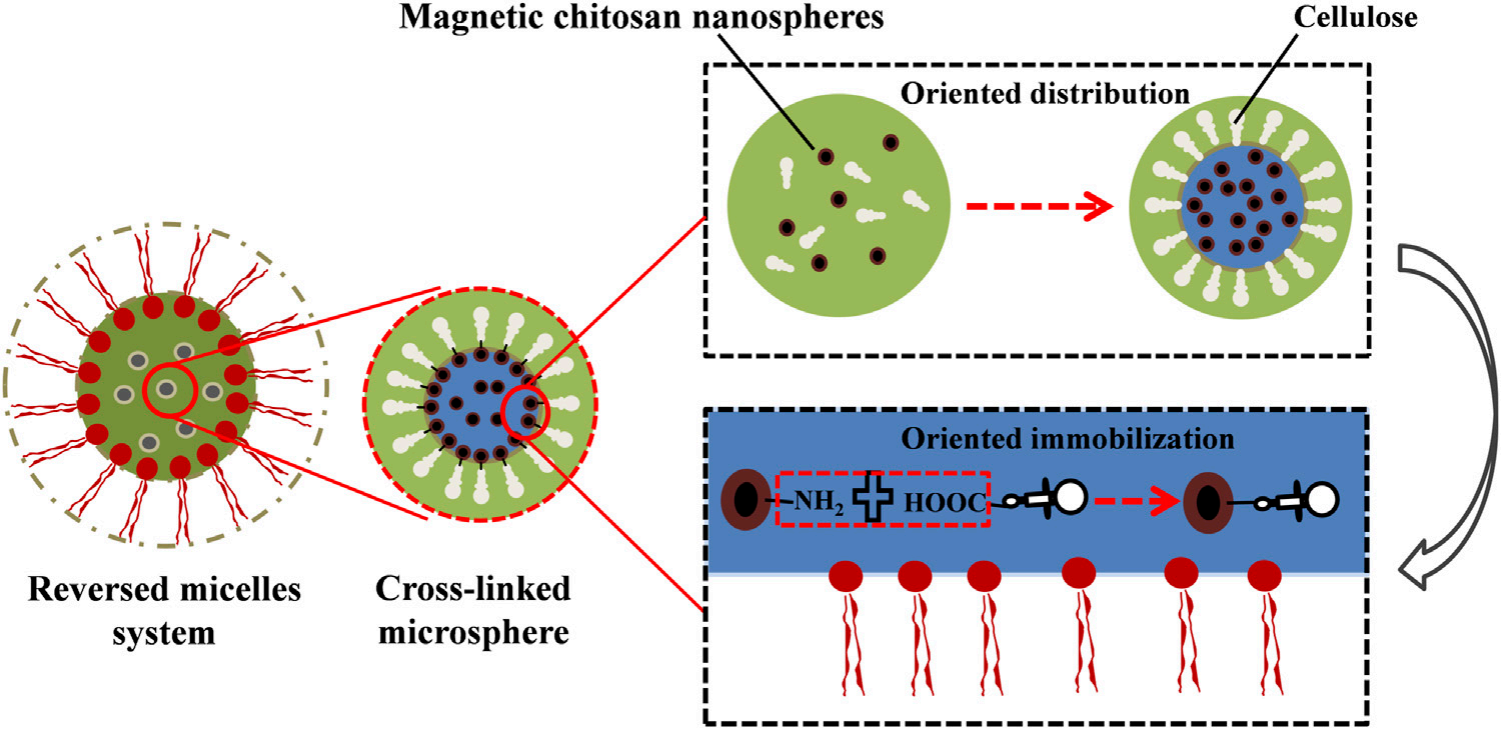
The oriented immobilization diagrammatic sketch of single-layer cellulase in the surfactant reversed micelles system, the “green” represents the internal “pool” of SRM system, “black” represents the magnetic chitosan microspheres (C-MNPs), “brown” represents the cross-linked microsphere.

**FIGURE 4:**
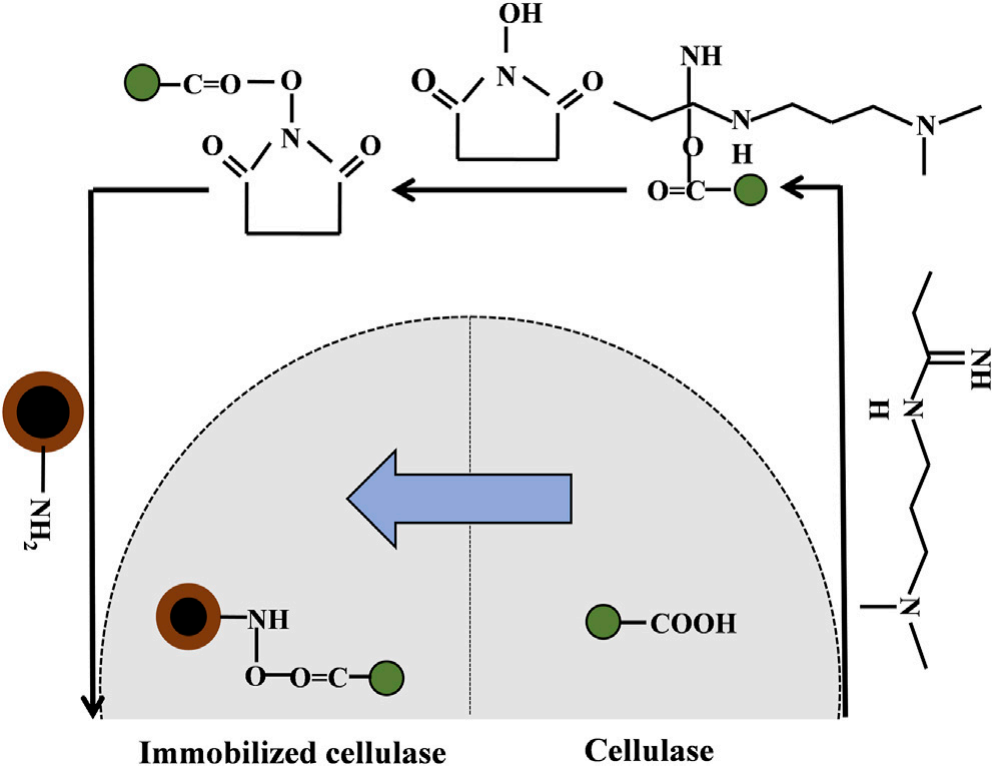
The oriented immobilization process of cellulase on magnetic nanoparticles.

Multipoint covalent attachment is likely the most effective strategy for immobilization, but it is difficult to allow the immobilization of enzymes at a well-defined position since the proteins are usually attached to the solid surface by uncontrolled chemical bonds (Barbosa et al., 2015; Hernandez and Fernandez-Lafuente, 2011; Li et al., 2016). The uncontrolled conformational changes were caused by random immobilization, which may lead to a significant loss of enzyme activity, and the disordered enzyme orientation may also reduce the accessibility of the substrate to functional sites (Orellana et al., 2018; Steen Redeker et al., 2013; Yu et al., 2012). However, the hydrophilic cellulase will be dissolved in the SRM system due to the existence of surfactants, which can maintain the activity of the enzyme and prevent the toxic effects of organic solvents (Tao et al., 2013). The active centers of cellulase molecules are usually clefts, which provide a different microenvironment (Zhang et al., 2015) because the structures of cellulase active centers are mainly composed of eight kinds of amino acids (tryptophan, tyrosine, histidine, phenylalanine, aspartic acid, glutamic acid, and arginine). Aromatic amino acids and some polar amino acids appeared more frequently, such as tryptophan, tyrosine, histidine, aspartate, asparagine and arginine, most of which are hydrophobic tryptophan and phenylalanine residues, especially the tryptophan which has the highest content and plays an important role in the recognition and binding of enzyme molecules and substrate (Zhang et al., 2015). Hydrophobic active centers are conducive to the combination of catalyzed groups of cellulase and substrates. When the specific substrate is close to the active centers, a change in the conformation of the cellulase molecule can be induced so that the reaction groups of the enzyme active centers and substrate are aligned correctly. Meanwhile, the molecular orbitals between the reaction groups of the active centers are strictly located in the right direction for easier enzymatic reactions. Therefore, cellulase is distributed in the W/O interface, and the catalytic active center is toward the organic solvent and the other side toward the “pool”. Moreover, the addition of surfactants can enhance the aggregation effect of cellulase on the W/O interface, and the existence of a crosslinking agent promotes the covalent crosslinking of enzyme molecules. The catalytic activity centers of the cross-linked microspheres are distributed uniformly and toward the outside, which solves the challenge of the uncontrollable attachment sites of the cellulase molecules in the immobilization process (Li et al., 2016; Steen Redeker et al., 2013; Yu et al., 2012). In the SRM system, the hydrophobic active molecules are exposed to the outside, which is beneficial for the further combination of immobilized cellulase and lignocellulosic substrates. However, the immobilized sites of cellulase molecules remain stochastic and heterogeneous, which may lead to covalent binding between the carriers and the active center of the enzyme and further cause ineffective immobilization and enzymatic reactions (Li et al., 2016). Therefore, to achieve oriented immobilization and improve the recycling times of cellulase, C-MNPs can be used as carriers as shown in Figure 3. This method can effectively prevent the ineffectiveness of cellulase immobilization. In this process, glutaraldehyde is used as the crosslinking agent, and EDC and N-hydroxysuccinimide are the coupling agents (Figure 4). In the W/O system, the free carboxyl group (-COOH) in the adsorption zone of the cellulase molecules can realize covalent binding with a large number of amino terminal catalytic residues of chitosan molecules (Fan et al., 2016). The process cannot destroy the catalytic center of cellulase, and the exposed catalytic clefts increase the effective attachment of immobilized cellulase to solid substrates, which further promotes enzymatic hydrolysis. Therefore, the oriented immobilization of enzymes is obtained in the SRM system, which can prevent nonproductive combinations effectively and further promote enzymatic hydrolysis.

## Conclusion

Cellulase plays an important role in the production of fuel ethanol by the enzymatic hydrolysis of lignocellulose, and the immobilization of cellulase on the nanocarriers is an effective way to improve hydrolysis efficiency. However, the nanocarrier structure characteristics, solid-solid contact obstacles, external diffusion resistance, limited recycling frequency of nanocarriers, and nonproductive combination of enzyme active centers restricted the further improvement of hydrolysis efficiency in the complex multiphase system. Surfactants can promote the enzymatic hydrolysis of lignocellulose and play an important role in the preparation of nanocarriers. The special SRM system caused by the amphiphilicity in the oil-water reaction system can provide effective protection to obtain the immobilization of single-layer cellulase, which can effectively prevent the immobilization of cellulase and increase the effective attachment of immobilized cellulase and solid substrates.
